# Simultaneous Characterization of Sympathetic and Cardiac Arms of the Baroreflex through Sequence Techniques during Incremental Head-Up Tilt

**DOI:** 10.3389/fphys.2016.00438

**Published:** 2016-09-29

**Authors:** Andrea Marchi, Vlasta Bari, Beatrice De Maria, Murray Esler, Elisabeth Lambert, Mathias Baumert, Alberto Porta

**Affiliations:** ^1^Department of Electronics Information and Bioengineering, Politecnico di MilanoMilan, Italy; ^2^Department of Emergency and Intensive Care, San Gerardo HospitalMonza, Italy; ^3^Department of Biomedical Sciences for Health, University of MilanMilan, Italy; ^4^IRCCS Fondazione Salvatore MaugeriMilan, Italy; ^5^Human Neurotransmitter Laboratory, Baker IDI Heart and Diabetes InstituteMelbourne, VIC, Australia; ^6^School of Electrical and Electronic Engineering, University of AdelaideAdelaide, SA, Australia; ^7^Department of Cardiothoracic, Vascular Anesthesia and Intensive Care, IRCCS Policlinico San DonatoMilan, Italy

**Keywords:** heart rate variability, arterial pressure variability, muscle sympathetic nerve activity, autonomic nervous system, cardiovascular control

## Abstract

We propose a sympathetic baroreflex (sBR) sequence method for characterizing sBR from spontaneous beat-to-beat fluctuations of muscle sympathetic nerve activity (MSNA) and diastolic arterial pressure (DAP). The method exploits a previously defined MSNA variability quantifying the fluctuations of MSNA burst rate. The method is based on the detection of MSNA and DAP sequences characterized by the contemporaneous DAP increase and MSNA decrease or *vice versa*. The percentage of sBR sequences (SEQ%_sBR_) was taken as an indication of the degree of sBR solicitation and the average slope of the regression lines in the (DAP, MSNA) plane was taken as sBR sensitivity (sBRS_SEQ_) and expressed in bursts^.^s^−1.^mmHg^−1^. sBRS_SEQ_ was compared to a more traditional estimate based on the baroreflex threshold analysis (sBRS_BTA_). An incremental head-up tilt protocol, carried out in 12 young healthy subjects (age: 20–36 yr, median = 22.5 yr, 9 females) sequentially tilted at 0, 20, 30, 40, 60° table inclinations, was utilized to set the sBR sequence method parameters. Traditional sequence analysis was exploited to estimate cardiac baroreflex (cBR) sensitivity (cBRS_SEQ_) and percentage of cBR sequences (SEQ%_cBR_). The head-up tilt induced the progressive increase of SEQ%_sBR_ and SEQ%_cBR_ and gradual decrease of both sBRS_SEQ_ and cBRS_SEQ_, thus suggesting the gradual rise of the sBR and cBR solicitations and the progressive reduction of their effectiveness with the stimulus. sBRS_SEQ_ was significantly associated with sBRS_BTA_. sBRS_SEQ_ and cBRS_SEQ_ were significantly correlated as well as SEQ%_sBR_ and SEQ%_cBR_, even though the correlation was not strong, thus suggesting a certain degree of independence between the baroreflex arms. The proposed sBR sequence approach provides a dynamical characterization of the sBR alternative to more traditional static pharmacological and nonpharmacological methods and fully homogenous with the cBR sequence technique.

## Introduction

Baroreflex plays a central role in arterial pressure (AP) homeostasis (Robertson et al., 2012) by triggering modifications of many physiological variables including e.g., heart period (HP), sympathetic activity and peripheral resistances in the attempt to buffer AP variations. The arm of the baroreflex acting upon HP, usually referred to as cardiac baroreflex (cBR), is one of the most actively studied (Hunt et al., 2001). The quantitative estimation of the cBR sensitivity (cBRS) was based on the observation that HP lengthens (or shortens) in response to the AP rise (or fall) induced by a quick, intravenous, administration of a vasoactive drug (Smyth et al., 1969; Pickering et al., 1972). cBRS is measured as the slope of the linear regression of HP on systolic AP (SAP) following the pharmacological challenge. Parallel increases or decreases in both HP and SAP, usually referred to as cBR sequences, were found even in absence of pharmacologically induced SAP changes when spontaneous HP and SAP variations were contemporaneously observed on a beat-to-beat basis (Bertinieri et al., 1985). This observation allowed the estimation of the cBRS in more physiological conditions from spontaneous variations of HP and SAP as the average slope of the regression lines computed over the cBR sequences.

cBR is just one arm of the baroreflex. Indeed, since modifications of AP lead to changes of the sympathetic activity that restore AP, a sympathetic arm of the baroreflex has been presumed (Sundlöf and Wallin, 1978). The sympathetic baroreflex (sBR) has been characterized in humans by exploiting microneurographic recordings of integrated muscle sympathetic nerve activity (MSNA). Since the likelihood of a MSNA burst increases while diastolic AP (DAP) decreases, the slope of the linear regression of the percentage of heart beats associated to a MSNA burst at a given DAP value on the DAP value has been proposed to estimate the sBR sensitivity (sBRS) (Sundlöf and Wallin, 1978; Rudas et al., 1999; Kienbaum et al., 2001).

Despite the relevance of evaluating simultaneously sBR and cBR, few studies so far assessed contemporaneously the two different aspects of the same control reflex (Rudas et al., 1999; O'Leary et al., 2003; Dutoit et al., 2010; Taylor et al., 2015). This is the consequence of the difficulties in defining a common framework to assure a homogenous characterization of both sBR and cBR. This common framework is difficult to be set when using methods exclusively based on pharmacological interventions (Rudas et al., 1999; Dutoit et al., 2010) or spontaneous physiological variations (O'Leary et al., 2003; Taylor et al., 2015). Indeed, when the modified Oxford method (Ebert and Cowley, 1992) was utilized to compute both sBRS and cBRS (Rudas et al., 1999; Dutoit et al., 2010), their estimates might have different reliability given that at high DAP the likelihood of detecting MSNA burst is null, thus clipping the useful range of AP variations compared to that exploited for the cBRS estimate. On the other hand, when the estimation of both sBRS and cBRS was based on spontaneous variations (O'Leary et al., 2003; Taylor et al., 2015), the comparison is again weak because a dynamical method testing the solicitation of cBR through the selection of sequences of cBR origin (Bertinieri et al., 1985) was compared to a static method searching for the mere association between binned values of DAP and the occurrence of MSNA burst without assuring that a positive DAP variation produces a negative MSNA burst rate change or *vice versa*.

The issue of setting a common framework for the contemporaneous assessment of sBRS and cBRS might be easily tackled if baroreflex sequence analysis could be extended to the MSNA and DAP variabilities. Unfortunately, this extension has not been proposed yet due to the unsuitability of the definition of the traditional MSNA variability series. Indeed, since the traditional MSNA variability series is an uncalibrated signal obtained from the integrated MSNA via low-pass filtering preserving exclusively the MSNA oscillations in the range of cardiovascular variability (i.e., from 0 to 0.5 Hz), it cannot provide sBRS estimates with suitable units (i.e., bursts^.^s^−1.^mmHg^−1^; Saul et al., 1990; Pagani et al., 1997; Nakata et al., 1998; Taylor et al., 1998; Cooke et al., 1999; Furlan et al., 2000; Kamiya et al., 2005; Ryan et al., 2011) and requires normalization procedures to avoid the dependence of results on factors that are not under control of the investigator (e.g., proximity of the bundle to the recording electrode). Also some classical static methods based on the computation of amplitude or area of the MSNA bursts and its association with binned values of DAP (Sundlöf and Wallin, 1978) require normalization procedures (e.g., the largest MSNA burst amplitude or area is assigned to an arbitrary value and the amplitude or area of all other bursts is expressed in proportion to this value; Kienbaum et al., 2001). This issue can be circumvented by the recent introduction of a calibrated MSNA variability signal (Marchi et al., 2016) focusing on modulations of the burst rate instead of variations of the amplitude of the burst.

The aim of this study is to propose a sBR sequence method for computing sBRS, optimize the parameters of the method by using a graded orthostatic challenge and compare sBRS with cBRS. The study exploits the recently defined calibrated MSNA variability (Marchi et al., 2016) in association with spontaneous DAP variations to compute sBRS, while cBRS is computed over spontaneous HP and SAP variability via the traditional cBR sequence method (Bertinieri et al., 1985). sBRS and cBRS are calculated during the baroreflex unloading induced by an incremental head-up tilt protocol in healthy volunteers (Lambert et al., 2008) imposing a sympathetic activation (Marchi et al., 2016) and a reduction of cBRS (Cooke et al., 1999; Porta et al., 2016) and sBRS (Ichinose et al., 2006; Barbic et al., 2015; Marchi et al., 2015) proportional to the magnitude of the challenge. This study hypothesizes that sBRS decreases in proportion to the central hypovolemia induced by incremental head-up tilt and tests *a posteriori* that the proposed sBR sequence method can monitor this decrease. The sBR sequence method is compared to the baroreflex threshold analysis (BTA), i.e., the traditional static method for the characterization of sBR based on the likelihood of finding a MSNA bust in correspondence of a given DAP value (Sundlöf and Wallin, 1978; Kienbaum et al., 2001; Hart et al., 2010).

## Methods

### Experimental protocol and data acquisition

The study comprised 12 healthy subjects (age from 20 to 36 yr, median = 22.5 yr; BMI: from 18.6 to 28.4 kg^.^m^−2^, median = 24.2 kg^.^m^−2^; 9 females). The study protocol was approved by the Alfred Hospital Ethics Review Committee (n. 144/06) and conformed to the relevant guidelines of the National Health and Medical Research Council of Australia and to the principles of the Declaration of Helsinki for medical research involving humans. All subjects provided written informed consent. The head-up tilt protocol was fully described in Lambert et al. (2008). Briefly, all subjects were tested in the morning, after a light breakfast. Caffeine and alcohol intake was restricted from 7:00 p.m. on the evening before the study. The radial artery was cannulated percutaneously (3F, 5 cm, Cook catheter) to enable invasive AP monitoring. A lead III electrocardiogram (ECG) was recorded via a single lead ECG amplifier (ADInstruments, Castle Hill, NSW, Australia). Respiration movements were monitored via a piezoelectric device (ADInstruments, Castle Hill, NSW, Australia). Multiunit sympathetic nerve firing rates in postganglionic fibers distributed to the skeletal muscle vasculature were recorded by using clinical microneurography via the IOWA Nerve Traffic Analyzer (model 662C-3, Department of Bioengineering, University of Iowa, Iowa; Lambert et al., 2008). After locating the common peroneal nerve, a tungsten microelectrode (FHC, Bowdoinham, Maine) was inserted percutaneously and adjusted until satisfactory spontaneous MSNA was observed in accordance with previously described criteria (Lambert et al., 2008). After instrumentation subjects were allowed to rest for at least 30 min. Then, subjects were sequentially tilted to angles 0, 20, 30, 40, and 60° (T0, T20, T30, T40, and T60, respectively) for 10 min at each angle. The head-up tilt test started from the horizontal position and was incremental with respect to the previous tilt table inclination. ECG, AP, respiratory movements and MSNA were continuously measured at each tilt angle. The raw MSNA signal was band-pass filtered (700–2000 Hz), amplified, rectified, and integrated (time constant of 0.1 s) to obtain integrated MSNA. ECG, AP, respiratory movements and integrated MSNA, were digitized at 1000 Hz using a PowerLab system (model ML785/8SP, ADInstruments, Castle Hill, NSW, Australia) and stored for off-line analysis. From the respiratory movement signal the respiratory rate was computed. Out of all recordings obtained from the 12 subjects, signals were of insufficient quality or the tilt protocol was not completed, respectively, for one subject during T30 and T40 as well as 5 subjects during T60.

### Extraction of the beat-to-beat variability series

**Figure 1:**
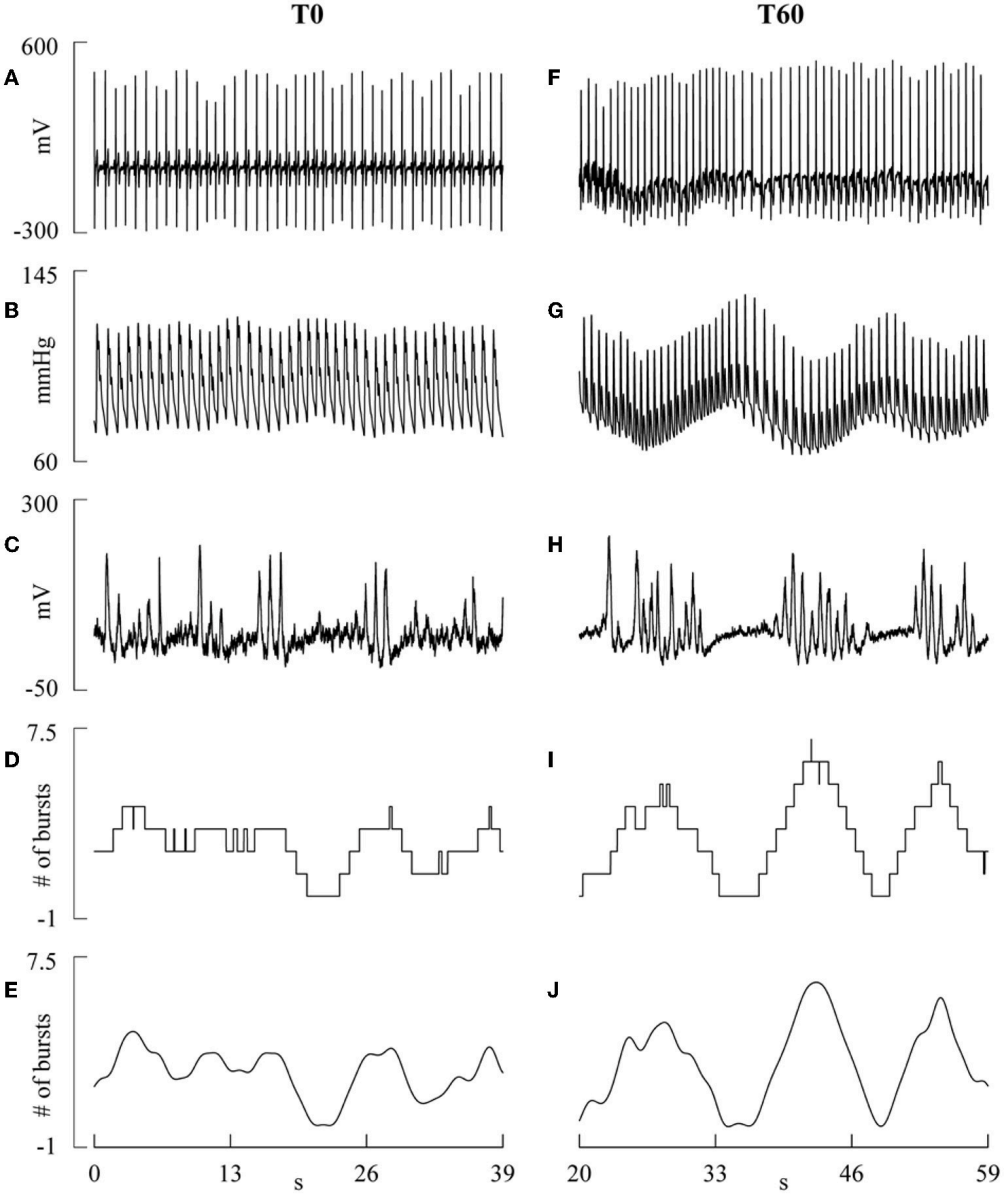
**The line plots show examples of ECG, invasive AP, integrated MSNA, step-wise burst-count MSNA and low-pass burst-count MSNA during T0 (A–E) and T60 (F–J)**. The slow oscillations in the low-pass burst-count MSNA occurring in phase opposition to slow changes of SAP and DAP visible on AP are more evident during T60 than T0, while faster oscillations are more present during T0.

The R-wave peaks were detected using a traditional method based on a threshold on the first derivative. The jitters in locating the R-wave peak were minimized using parabolic interpolation, thus achieving a time resolution smaller than 1 ms. The temporal distance between two consecutive parabolic apexes was taken as HP. The maximum of AP inside the *i*-th HP was defined as the *i*-th SAP value, where *i* is the cardiac beat counter. The *i*-th DAP was computed as the minimum of the AP preceding the *i*-th SAP. The occurrences of R-wave peaks and the positions of SAP and DAP fiducial points were carefully checked to avoid erroneous detections or missed beats. The MSNA variability was extracted according to the procedure proposed in Marchi et al. (2016). Briefly, first MSNA bursts were detected from the integrated MSNA signal by using an adaptive thresholding method capable of following on a beat-to-beat basis the baseline wandering and physiological variations of the MSNA burst amplitude (Diedrich et al., 2009). The detection procedure accounted for the latency from the AP sensing to possible MSNA response (Sundlöf and Wallin, 1978; Wallin et al., 1994; Hamner and Taylor, 2001) by searching the MSNA burst in a time window ranging from 0.9 to 1.7 s starting from the first R-wave peak delimiting the current HP (Kienbaum et al., 2001; Diedrich et al., 2009). After having detected the MSNA burst associated to each R-wave peak, the MSNA bursts were counted over a moving time window of 5 s that was advanced in steps of 1 ms along the recording, thus obtaining a step-wise burst-count MSNA signal. Finally, the step-wise burst-count MSNA signal was low-pass filtered with a finite impulse response filter with a cut-off frequency of 0.5 Hz (8000 coefficients). An example of ECG, AP, integrated MSNA, step-wise burst-count MSNA, and low-pass filtered burst-count MSNA signal recorded from the same subject during T0 and T60 is given in Figure 1. The low-pass filtered burst-count integrated MSNA signal was downsampled once per cardiac beat at the occurrence of the first R-wave peak delimiting the *i*-th HP, thus obtaining a burst-count MSNA beat-to-beat variability series synchronous with HP, SAP, and DAP series. Each value of the burst-count MSNA variability series was divided by the length of the moving time window (i.e., 5 s), yielding the MSNA burst rate variability expressed in bursts^.^s^−1^ and briefly indicated as MSNA variability in the following. The choice of the duration of the time window (i.e., 5 s) is the result of an optimization process. We computed the linear regression of the power of the variability of the MSNA burst rate in the frequency band centered about 0.1 Hz (from 0.04 to 0.15 Hz), as assessed via autoregressive power spectral analysis (Pagani et al., 1997), on the sine of tilt table angle as a function of the window length. We tested window lengths of 3, 5, 10, 15, 20 s and we found out that the length of 5 s maximized the correlation coefficient of the linear regression (respectively *r* = 0.426, *p* = 1.82 × 10^−3^; *r* = 0.441, *p* = 1.19 × 10^−3^; *r* = 0.419, *p* = 2.20 × 10^−3^; *r* = 0.245, *p* = 8.35 × 10^−2^; *r* = 0.384, *p* = 5.39 × 10^−3^). Longer windows smoothed the oscillations about 0.1 Hz probably because it is highly likely that periods of MSNA silence were included in any window. Shorter windows (i.e., 3 beats) produced some smoothing as well, mainly as a consequence of the limited number of MSNA bursts detected in any window and the more important transitions from a MSNA burst rate level and its adjacent one compared to those relevant to longer window lengths.

**Figure 2:**
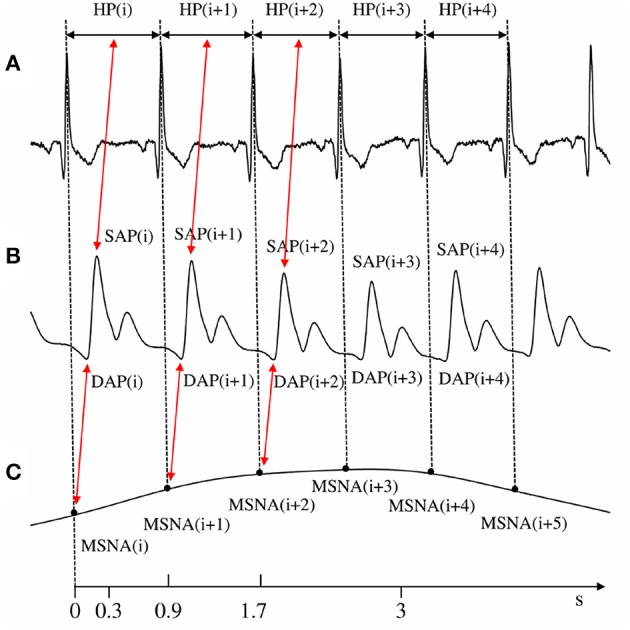
**The conventions for the construction of HP, SAP, DAP, and MSNA variability series from ECG (A), AP (B), and low-pass burst-count MSNA (C) are shown**. The associations between HP and SAP values, exploited for the computation of cBRS_SEQ_ and between MSNA variability and DAP utilized for the computation of sBRS_SEQ_, are reported as well. The time axis reports the lower and upper limits of the cBR and sBR latencies (i.e., 0.3–3.0 s and 0.9–1.7 s, respectively) assumed in this study.

**Figure 3:**
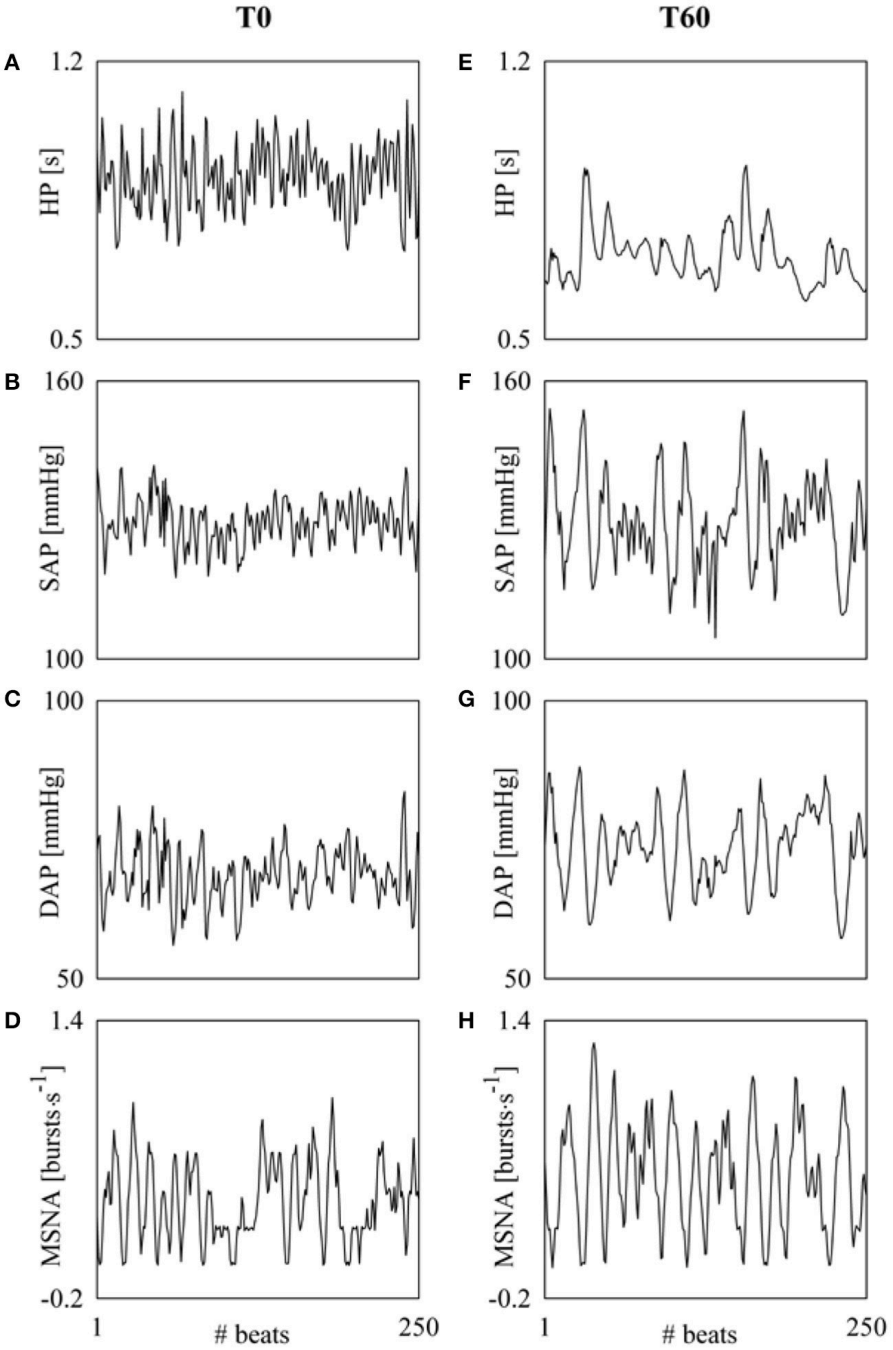
**The line plots show examples of HP, SAP, DAP, and MSNA beat-to-beat variability recorded during T0 (A–D) and T60 (E–H)**. Fast dynamics at the respiratory rate are more visible in HP and SAP series during T0 **(A,B)** and in SAP during T60 **(F)**. DAP and MSNA series **(C,D,G,H)** are dominated by rhythms at frequency slower than respiration.

Figure 2 provides a summary of the conventions used in this study for numbering the HP, SAP, DAP, and MSNA burst rate measurements. HP, SAP, DAP, and MSNA variability series of *N* = 256 consecutive values were randomly selected within each experimental condition. If non-stationarities, such as very slow drifting of the mean or sudden changes of the variance, were evident despite the linear detrending, the random selection was repeated. Figure 3 shows examples of the beat-to-beat HP, SAP, DAP, and MSNA variability series derived from the same subject as in Figure 1 during T0 and T60. The mean and the variance of HP, SAP, DAP, and MSNA variability series were calculated, indicated as μ_HP_, $\sigma_{\text{HP}}^{2}$, μ_*SAP*_, $\sigma_{\text{SAP}}^{2}$, μ_DAP_, $\sigma_{\text{DAP}}^{2}$, μ_MSNA_, and $\sigma_{\text{MSNA}}^{2}$, and expressed as ms, ms^2^, mmHg, mmHg^2^, mmHg, mmHg^2^, bursts^.^s^−1^, and bursts^2.^s^−2^, respectively.

### cBR sequence method

cBRS estimation was based on cBR sequence method (Bertinieri et al., 1985) as implemented in Porta et al. (2000). This technique relies on the search for spontaneous sequences characterized by the contemporaneous increase or decrease of HP and SAP. The sequences are composed by three consecutive HPs and SAPs. A spontaneous cBR sequence was considered to be meaningful if it matched the following prerequisites (Laude et al., 2004): (1) the absolute value of the total HP variation was larger than 5 ms (i.e., |ΔHP| > 5 ms); (2) the absolute value of the total SAP variation was larger than 1 mmHg (i.e., |ΔSAP| > 1 mmHg); (3) the linear correlation coefficient computed in the [SAP(*i*), HP(*i*+τ_HP−SAP_)] plane over a given cBR sequence, r_HP−SAP_, was larger than 0.85, where τ_*HP*−*SAP*_ represents the lag between HP and SAP. Two alternative methods to fix τ_HP−SAP_ were tested: (i) τ_HP−SAP_ = 0 beats (Figure 2) to pick up the fast vagal arm of the cBR capable of modifying HP in reaction of SAP changes occurring within the same HP (Eckberg, 1976; Baselli et al., 1994); (ii) τ_HP−SAP_ was optimized in the set {0,1,2,3} in keeping with the maximum of the HP-SAP cross-correlation function. The τ_HP−SAP_ optimization range was selected according to the minimum and maximum cBR latency set to 0.3 and 3.0 s, respectively (Eckberg, 1976; Porta et al., 2011): indeed, the association between SAP(*i*) and HP(i+τ_HP−SAP_) due to cBR should be negligibly present with τ_HP−SAP_ > 3 (Figure 2). The slope of the regression line was calculated for each cBR sequence and subsequently averaged over all sequences. This average was taken as an estimate of cBRS, labeled as cBRS_SEQ_ and expressed in ms^.^mmHg^−1^. The percentage of cBR sequences with respect to all sequences was computed as well and indicated as SEQ%_cBR_. SEQ%_cBR_ ranges between zero and 100 where zero indicates the absence of cBR sequences, while 100 indicates that all possible HP–SAP sequences are of cBR origin. While cBRS_SEQ_ is taken as a measure of the cBR effectiveness, SEQ%_cBR_ is taken as a measure of the degree of cBR involvement.

### sBR sequence method

sBRS_SEQ_ was estimated with the same logic as cBRS_SEQ_ over the MSNA and DAP beat-to-beat variability series. This application was made possible by the exploitation of the MSNA series defined in Marchi et al. (2016) expressing the MSNA variability in bursts^.^s^−1^. Specifically, the sBR sequence technique relied on the search for spontaneous sequences characterized by the contemporaneous increase of MSNA burst rate and decrease of DAP or *vice versa*. The prerequisites relevant to linear correlation coefficient in the [DAP(*i*), MSNA(*i*+τ_MSNA−DAP_)] plane, r_MSNA−DAP_, and the total variation of DAP, |ΔDAP|, reflected the usual setting of the cBR sequence analysis (i.e., |r_MSNA−DAP_| > 0.85) and the typical bin size set in more traditional methods for sBRS estimation from spontaneous DAP variability (i.e., |ΔDAP| > 1 mmHg; Hart et al., 2010). Also the length of the sBR sequences was maintained equal to that of cBR sequences and corresponded to three consecutive DAPs and MSNA burst rate values. τ_MSNA−DAP_, representing the lag between MSNA burst rate and DAP expressed in beats, was optimized in the set {0,1,2,3} to fully account for the latency of sBR in modifying MSNA burst rate after sensing DAP (Kienbaum et al., 2001; Diedrich et al., 2009; Figure 2). The selected range of τ_MSNA−DAP_ is in keeping with that selected for cBR characterization and sBR latency. The prerequisite relevant to the absolute value of the MSNA burst rate variation, |ΔMSNA|, was optimized as well. We tested different minimal levels of |ΔMSNA| corresponding to the prerequisite of |ΔMSNA| > *x* bursts^.^s^−1^, where *x* was initially set to 0.0 bursts^.^s^−1^and, then, increased in steps of 0.05 bursts^.^s^−1^ up to 0.5 bursts^.^s^−1^. The upper limit of 0.5 bursts^.^s^−1^ was chosen according to the maximum |ΔMSNA| observed in our protocol given the adopted sequence length. The slope of the regression line was calculated for each sBR sequence and subsequently averaged over all sequences. This average was taken as an estimate of sBRS, labeled as sBRS_SEQ_ and expressed in bursts^.^s^−1.^mmHg^−1^. The percentage of sBR sequences with respect to all sequences was computed as well and indicated as SEQ%_sBR_. SEQ%_sBR_ ranges between zero and 100 where zero indicates the absence of sBR sequences, while 100 indicates that all possible MSNA-DAP sequences are of baroreflex origin. While sBRS_SEQ_ is taken as a measure of the sBR effectiveness, SEQ%_sBR_ is taken as a measure of the degree of sBR involvement.

### BTA method

BTA is based on the computation of the percentage of MSNA bursts associated with a given level of DAP (Sundlöf and Wallin, 1978; Kienbaum et al., 2001; Hart et al., 2010). The size of the DAP bin was fixed to 1 mmHg (Hart et al., 2010). More specifically, the percentage of heart beats associated to a given MSNA burst was plotted as a function of the mean DAP in the assigned bin and the slope of the regression line in this plane was taken as an estimate of the sBRS and indicated as sBRS_BTA_ in the following. To account for the sBR latency the MSNA burst was searched for in a time window ranging from 0.9 to 1.7 s starting from the R-wave peak (Kienbaum et al., 2001; Diedrich et al., 2009). Since the likelihood to find a MSNA bust decreases with DAP, sBRS_BTA_ is smaller than zero.

### Statistical analysis

We carried out the Bartlett test, or the Levene test when appropriate, to check for homoscedasticity of the computed parameters in the various experimental sessions. If the null hypothesis of homogeneity of variance was rejected, we log-transformed the absolute value of the parameter and the test for homoscedasticity was repeated. One-way analysis of variance (Dunnett's test for multiple comparisons), or Kruskal–Wallis one-way analysis of variance on ranks when appropriate (Dunn's test for multiple comparisons), was applied to test the significance of the changes of time domain parameters compared to T0. Paired *t*-test, or Wilcoxon signed rank test when appropriate, was applied to check the difference between SEQ%_cBR_ and SEQ%_sBR_ when data were pooled together regardless of the experimental condition. Linear regression analysis was carried out to check the presence of a linear relation of cBRS_SEQ_, SEQ%_cBR_, sBRS_*SEQ*_, and SEQ%_sBR_ on the sine of the tilt table angles (the sine is taken because the stimulus is proportional to the component of the gravity parallel to the tilt table). The Pearson product moment correlation coefficient, *r*, was computed and the null hypothesis of a slope equal to zero (i.e., no linear relationship) was tested. If the null hypothesis of homoscedasticity was rejected, the result of linear regression analysis over the original values was checked after the log-transformation of the absolute values of the parameter. In the case of sBRS_SEQ_ and SEQ%_sBR_ the linear regression analysis was carried out as a function of the minimal |ΔMSNA| utilized to detect a meaningful sBR sequence and τ_MSNA−DAP_. Linear regression analysis of sBRS_BTA_ on sBRS_SEQ_ was performed to measure the degree of association between the nonpharmacological traditional method and the proposed method. A similar analysis was carried out between sBRS_SEQ_ and cBRS_SEQ_ and between SEQ%_sBR_ and SEQ%_cBR_ to assess the degree of association between cBR and sBR parameters. The Pearson product moment correlation coefficient, *r*, was computed and the null hypothesis of a slope equal to zero (i.e., no linear relationship) was tested after pooling together all subjects regardless of the experimental condition. If the null hypothesis of homoscedasticity was rejected of either of the variables or both, the result of linear regression analysis was checked after log-transforming the absolute values of the variable that did not pass the test for homogeneity of variance. Statistical analysis was carried out using a commercial statistical program (Sigmaplot, Systat Software, Inc, Chicago, IL, USA, ver.11.0). A *p* < 0.05 was considered statistically significant.

## Results

### Optimization of the parameters for spontaneous sBR sequence analysis

While the minimal |ΔDAP|, minimal |r_MSNA−DAP_|, and duration of the sBR sequence were set to |ΔDAP| = 1 mmHg, |r_MSNA−DAP_| = 0.85, and 3 beats respectively, the minimal |ΔMSNA| and τ_MSNA−DAP_ were optimized based on the analysis of the degree of linear association of SEQ%_sBR_ and sBRS_SEQ_ on tilt table inclination. The optimization of |ΔMSNA| and τ_MSNA−DAP_ is based on the assumption that the best setting of SEQ%_sBR_ and sBRS_SEQ_ should allow the detection of both a positive linear relation of SEQ%_sBR_ on the relevance of the baroreflex unloading, taken as an indication of the progressive solicitation of sBR with the magnitude of the challenge, and a positive linear relation of sBRS_SEQ_ with the sine of the tilt table angles, taken as a suggestion of a reduced effectiveness of sBR with the relevance of the stimulus. The correlation coefficients of the linear regression analysis of SEQ%_sBR_ on the sine of the tilt table inclination as a function of the minimal |ΔMSNA| and τ_MSNA−DAP_ are reported in Table 1. Results indicate that, regardless of the value of τ_MSNA−DAP_,SEQ%_sBR_ was significantly and positively related to the magnitude of the orthostatic challenge at the smallest values of the minimal |ΔMSNA| (i.e., below 0.4 bursts^.^s^−1^), thus suggesting the progressive sBR solicitation and the baroreflex origin of the detected sequences. The relation with the sine of the tilt table inclination disappeared at the highest values of the minimal |ΔMSNA| because the more restrictive prerequisite reduced the number of spontaneous sBR sequences to such a level that prevented the detection of a significant relation of SEQ%_sBR_ to tilt table angles. The correlation coefficients of the linear regression analysis of sBRS_SEQ_ on the sine of the tilt table inclination as a function of the minimal |ΔMSNA| and τ_MSNA−DAP_ are reported in Table 2. sBRS_SEQ_ exhibited a significant and positive association with the magnitude of the orthostatic challenge only at τ_MSNA−DAP_ = 0 beats and at the smallest values of the minimal |ΔMSNA| (i.e., below 0.15 bursts^.^s^−1^). Taking together the results shown in Tables 1, 2, a significant positive association of both SEQ%_sBR_ and sBRS_SEQ_ with the relevance of the orthostatic stimulus was observed at τ_MSNA−DAP_ = 0 beats and correlation coefficient picked the maximum at |ΔMSNA| > 0.0 bursts^.^s^−1^. Consequently, τ_MSNA−DAP_ = 0 and |ΔMSNA| > 0.0 bursts^.^s^−1^ were chosen as the most suitable setting for spontaneous sBR sequence analysis. The setting τ_MSNA−DAP_ = 0 was tested a posteriori by optimizing τ_MSNA−DAP_ in the set {0,1,2,3} according to the minimum of the MSNA-DAP cross-correlation function: the minimum was found at τ_MSNA−DAP_ = 0 in 86% of the analyses.

**Table 1:** **Correlation coefficient *r* of the regression line of SEQ%_sBR_ on the sine of the tilt table angles as a function of the minimal |ΔMSNA| and τ_MSNA−DAP_**.

	τ_MSNA−DAP_ = 0 beats	τ_MSNA−DAP_ = 1 beats	τ_MSNA−DAP_ = 2 beats	τ_MSNA−DAP_ = 3 beats
|ΔMSNA|>0.00 bursts.s^−1^	0.468^*^	0.482^*^	0.464^*^	0.521^*^
|ΔMSNA|>0.05 bursts.s^−1^	0.467^*^	0.479^*^	0.466^*^	0.520^*^
|ΔMSNA|>0.10 bursts.s^−1^	0.460^*^	0.472^*^	0.464^*^	0.510^*^
|ΔMSNA|>0.15 bursts.s^−1^	0.460^*^	0.464^*^	0.453^*^	0.494^*^
|ΔMSNA|>0.20 bursts.s^−1^	0.413^*^	0.441^*^	0.436^*^	0.466^*^
|ΔMSNA|>0.25 bursts.s^−1^	0.399^*^	0.423^*^	0.388^*^	0.408^*^
|ΔMSNA|>0.30 bursts.s^−1^	0.335^*^	0.380^*^	0.366^*^	0.373^*^
|ΔMSNA|>0.35 bursts.s^−1^	0.293^*^	0.293^*^	0.294^*^	0.331^*^
|ΔMSNA|>0.40 bursts.s^−1^	0.258	0.267	0.278	0.281
|ΔMSNA|>0.45 bursts.s^−1^	0.250	0.289	0.259	0.311
|ΔMSNA|>0.50 bursts.s^−1^	0.275	0.266	0.269	0.296

sBR, sympathetic baroreflex; SEQ%_sBR_, percentage of sBR sequences; |ΔMSNA|, absolute value of the total MSNA burst rate variation during a spontaneous sBR sequence; τ_MSNA−DAP_, lag between MSNA and DAP. The symbol

* indicates a p < 0.05.

**Table 2:** **Correlation coefficient *r* of the regression line of sBRS_SEQ_ on the sine of the tilt table angles as a function of the minimal |ΔMSNA| and τ_MSNA−DAP_**.

	τ_MSNA−DAP_ = 0 beats	τ_MSNA−DAP_ = 1 beats	τ_MSNA−DAP_ = 2 beats	τ_MSNA−DAP_ = 3 beats
|ΔMSNA|>0.00 bursts.s^−1^	0.302^*^	0.026	0.123	0.161
|ΔMSNA|>0.05 bursts.s^−1^	0.307^*^	0.015	0.128	0.160
|ΔMSNA|>0.10 bursts.s^−1^	0.297^*^	0.003	0.136	0.150
|ΔMSNA|>0.15 bursts.s^−1^	0.274	−0.030	0.100	0.147
|ΔMSNA|>0.20 bursts.s^−1^	0.238	−0.038	0.116	0.127
|ΔMSNA|>0.25 bursts.s^−1^	0.260	0.102	0.048	0.105
|ΔMSNA|>0.30 bursts.s^−1^	0.265	0.155	0.126	0.071
|ΔMSNA|>0.35 bursts.s^−1^	0.212	0.020	0.138	0.227
|ΔMSNA|>0.40 bursts.s^−1^	0.201	−0.029	0.126	0.263
|ΔMSNA|>0.45 bursts.s^−1^	0.275	0.084	0.230	0.278
|ΔMSNA|>0.50 bursts.s^−1^	0.269	0.184	0.227	0.272

sBR, sympathetic baroreflex; sBRS_SEQ_, sBR sensitivity computed with the sBR sequence method; |ΔMSNA|, absolute value of the total MSNA burst rate variation during a spontaneous sBR sequence; τ_MSNA−DAP_, lag between MSNA and DAP. The symbol

* indicates a p < 0.05.

### Results of time domain indexes during incremental head-up tilt

Time domain parameters computed over HP, SAP, DAP, and MSNA variability were reported in Table 3. As a function of tilt table inclination, μ_HP_ gradually decreased showing a significant difference at T40 and T60 compared to T0. $\sigma_{\text{HP}}^{2}$, μ_SAP_ and μ_DAP_ remained stable, while $\sigma_{\text{SAP}}^{2}$ and $\sigma_{\text{DAP}}^{2}$ significantly increased during T60. We found that both μ_MSNA_ and $\sigma_{\text{MSNA}}^{2}$ increased with the magnitude of the postural challenge, thus suggesting an increase of tonic MSNA and its modulation about the mean value. Indeed, while μ_MSNA_ increased significantly during T40 compared to baseline, $\sigma_{\text{MSNA}}^{2}$ rose significantly during both T40 and T60.

**Table 3:** **Time domain parameters**.

**Index**	*T*0	*T*20	*T*30	*T*40	*T*60
μ_HP_ [ms]	1003 ± 147	927 ± 137	885 ± 129	806 ± 113^*^	688 ± 120^*^
$\sigma_{\text{HP}}^{2}$ [ms^2^]	5442 ± 3440	4553 ± 3652	4346 ± 2605	3005 ± 1817	2498 ± 939
μ_SAP_ [mmHg]	122.8 ± 21.4	123.0 ± 17.9	125.2 ± 20.4	124.2 ± 15.6	122.3 ± 16.7
$\sigma_{\text{SAP}}^{2}$ [mmHg^2^]	13.7 ± 9.2	11.8 ± 6.8	14.8 ± 8.9	21.9 ± 14.2	35.5 ± 22.2^*^
μ_DAP_ [mmHg]	67.6 ± 11.4	68.6 ± 11.1	70.0 ± 14.9	72.9 ± 11.3	75.5 ± 14.8
$\sigma_{\text{DAP}}^{2}$ [mmHg^2^]	10.5 ± 7.7	10.5 ± 5.7	10.3 ± 6.1	13.6 ± 7.2	23.4 ± 10.9^*^
μ_MSNA_ [bursts.s^−1^]	0.33 ± 0.09	0.37 ± 0.08	0.47 ± 0.13	0.50 ± 0.13^*^	0.45 ± 0.16
$\sigma_{\text{MSNA}}^{2}$ [bursts^2^.s^−2^]	0.033 ± 0.010	0.043 ± 0.015	0.044 ± 0.008	0.057 ± 0.018^*^	0.055 ± 0.021^*^

μ_HP_, HP mean; $\sigma_{\text{HP}}^{2}$, HP variance; μ_SAP_, SAP mean; $\sigma_{SAP}^{2}$, SAP variance; μ_DAP_, DAP mean; $\sigma_{DAP}^{2}$, DAP variance; μ_MSNA_, MSNA burst rate mean; $\sigma_{\text{MSNA}}^{2}$, MSNA burst rate variance; T0, T20, T30, T40, and T60, head-up tilt at 0, 20, 30, 40, and 60 degrees. Values are expressed as mean ± standard deviation. The symbol

* indicates a p < 0.05 vs. T0.

### cBR and sBR controls during incremental head-up tilt

**Figure 4:**
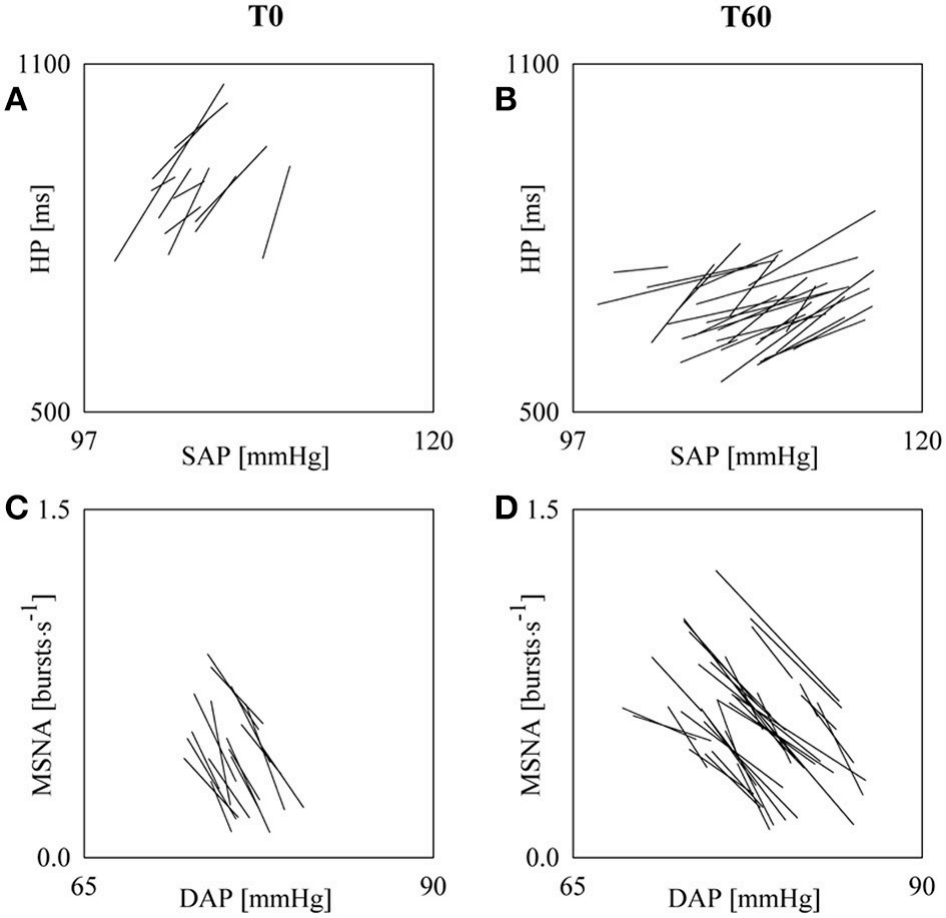
**The line plots show the linearly-interpolated spontaneous cBR (A,B) and sBR (C,D) sequences derived in a subject from SAP and HP series and from DAP and MSNA series respectively during T0 (A,C) and T60 (B,D)**. The analyses were carried out over SAP, HP, DAP, and MSNA series of 256 values. The larger number of cBR and sBR sequences, leading to larger SEQ%_cBR_ and SEQ%_sBR_, and the flatter slopes, leading to cBRS_SEQ_ and sBRS_SEQ_ closer to zero, are evident during T60 compared to T0.

Figure 4 shows examples of cBR and sBR sequence analyses carried out in a subject during T0 (Figures 4A,C) and T60 (Figures 4B,D). The panels show the linearly-interpolated cBR and sBR sequences whose mean slope is the cBRS_SEQ_ (Figures 4A,B) and sBRS_SEQ_ (Figures 4C,D). The slopes of the cBR sequences are positive (Figures 4A,B), while the slopes of the sBR sequences are negative (Figures 4C,D). During T60 (Figure 4B) the cBR sequences are flatter (i.e., the slopes are closer to zero) than during T0 (Figure 4A) and their number is larger. Since the analysis during T0 was carried out over series of the same length (i.e., 256 consecutive values) as during T60, also SEQ%_cBR_ was higher during T60 than T0. Indeed, SEQ%_cBR_ is 3.70 and 9.78 during T0 and T60, respectively and cBRS_SEQ_ is 35.56 and 14.83 ms^.^mmHg^−1^. The comparison of the results relevant to sBR sequences during T60 (Figure 4D) and T0 (Figure 4C) leads to the same conclusion. Indeed, sBR sequences are less steep during T60 (i.e., sBRS_SEQ_ is −0.120 and −0.075 bursts^.^s^−1.^mmHg^−1^ during T0 and T60, respectively) and their number increases (i.e., SEQ%_sBR_ is 5.39 and 12.68 during T0 and T60, respectively).

**Figure 5:**
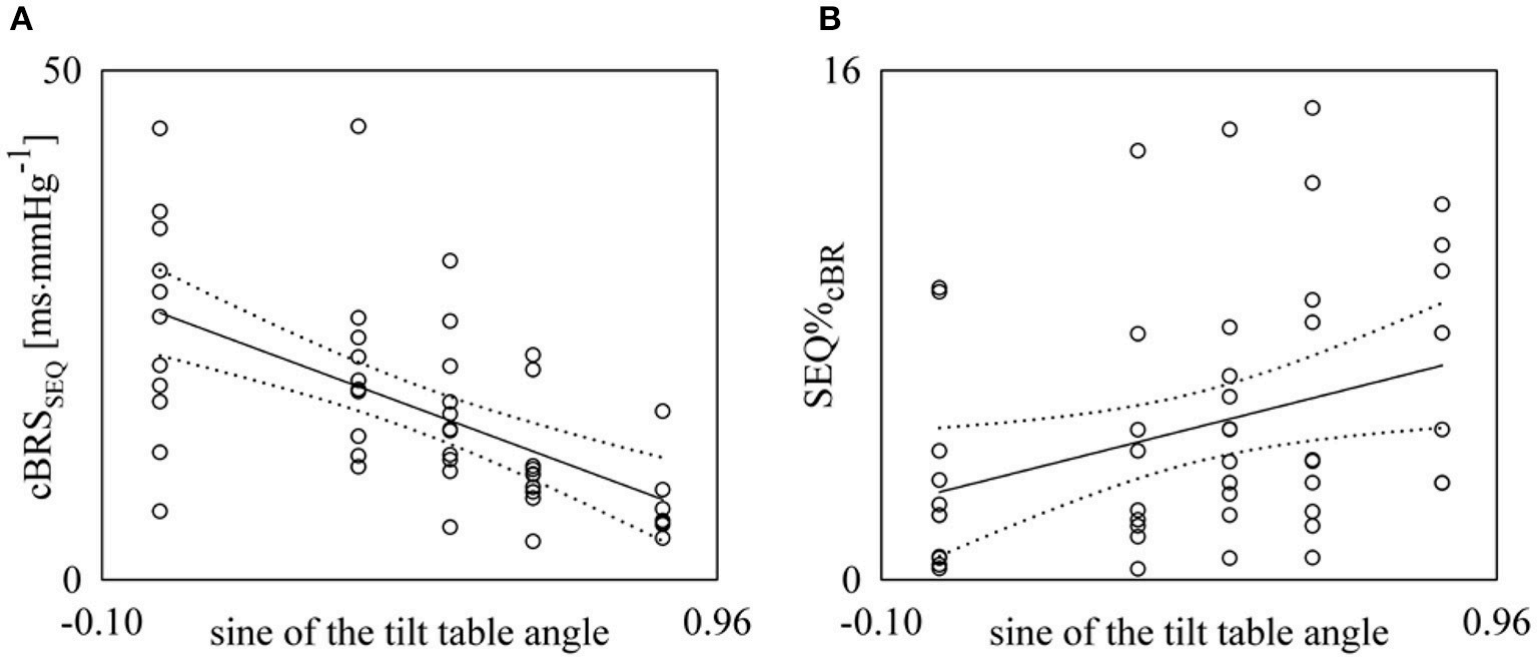
**Linear regression analyses of cBRS_SEQ_ (A) and SEQ%_cBR_ (B) on the sine of the tilt table angles during incremental orthostatic challenge**. Individual values (open circles) of cBRS_SEQ_ and SEQ%_cBR_ are shown as a function of the sine of the tilt table inclination. The linear regression (solid line) and its 95% confidence interval (dotted lines) are plotted as well if the slope of the regression line is significantly larger than zero with *p* < 0.05.

Figure 5 shows the individual values (open circles) of cBRS_SEQ_ (Figure 5A) and SEQ%_cBR_ (Figure 5B) as a function of the sine of the tilt table inclination. These results were obtained with τ_HP−SAP_ = 0. The regression line of the variable on the sine of the tilt table angles (solid line) and its 95% confidence interval (dotted lines) are plotted as well if the slope of the regression line is significantly larger than zero. A significant negative association of cBRS_SEQ_ (Figure 5A, *r* = −0.607, *p* = 2.93 × 10^−6^) and a significantly positive correlation of SEQ%_cBR_ (Figure 5B, *r* = 0.328, *p* = 2.02 × 10^−2^) with the relevance of the postural challenge were found. A slightly stronger association was obtained after optimizing τ_*HP*−*SAP*_ on individual basis: indeed, cBRS_SEQ_ was negatively associated with tilt table angles with *r* = −0.687 and *p* = 3.71 × 10^−8^, while SEQ%_cBR_ was positively associated with *r* = 0.454, *p* = 9.30 × 10^−4^.

**Figure 6:**
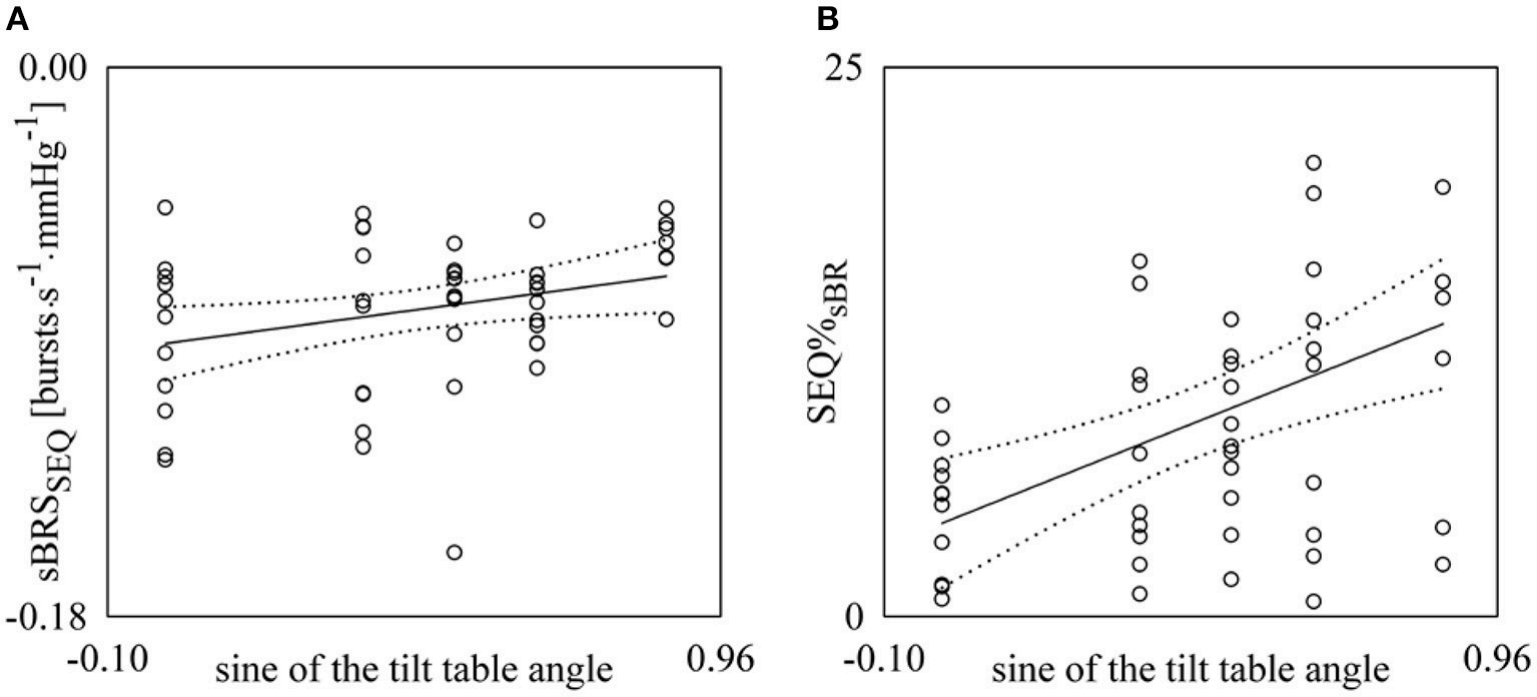
**Linear regression analyses of sBRS_SEQ_ (A) and SEQ%_sBR_ (B) on the sine of the tilt table angles during incremental orthostatic challenge**. Individual values (open circles) of sBRS_SEQ_ and SEQ%_sBR_ are shown as a function of the sine of the tilt table inclination. The linear regression (solid line) and its 95% confidence interval (dotted lines) are plotted as well if the slope of the regression line is significantly larger than zero with *p* < 0.05.

Figure 6 shows the individual values (open circles) of sBRS_SEQ_ (Figure 6A) and SEQ%_sBR_ (Figure 6B) as a function of the sine of the tilt table angle. According to the optimization procedure these results were obtained with τ_MSNA−DAP_ = 0 beats and |ΔMSNA| > 0.0 bursts^.^s^−1^. The regression line of the variable on the sine of the tilt table angles (solid line) and its 95% confidence interval (dotted lines) are plotted as well if the slope of the regression line is significantly larger than zero. Significant positive associations of sBRS_SEQ_ (Figure 6A, *r* = 0.302, *p* = 3.13 × 10^−2^) and SEQ%_sBR_ (Figure 6B, *r* = 0.468, *p* = 5.38 × 10^−4^) with the magnitude of the orthostatic challenge were found.

**Figure 7:**
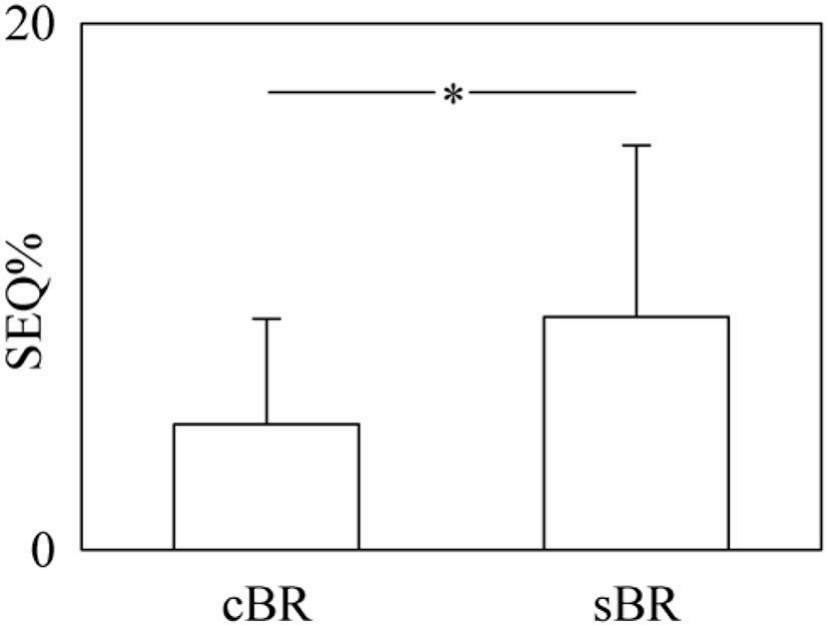
**The bar graph shows the percentage of sequences (SEQ%) as a function of the arm of the baroreflex (i.e., cBR and sBR)**. SEQ% values were pooled together regardless of the experimental condition. Values are given as mean plus standard deviation. The symbol ^*^ denotes a *p* < 0.05.

Figure 7 shows the percentage of sequences (SEQ%) as a function of the arm of the baroreflex (i.e., cBR and sBR). SEQ% values were pooled together regardless of the experimental condition. Values are reported as mean plus standard deviation. SEQ%_cBR_ was zero in one subjects during T0. SEQ%_cBR_ and SEQ%_sBR_ were 4.77 ± 3.99 and 8.84 ± 6.52, respectively. SEQ%_sBR_ was significantly higher than SEQ%_cBR_.

**Figure 8:**
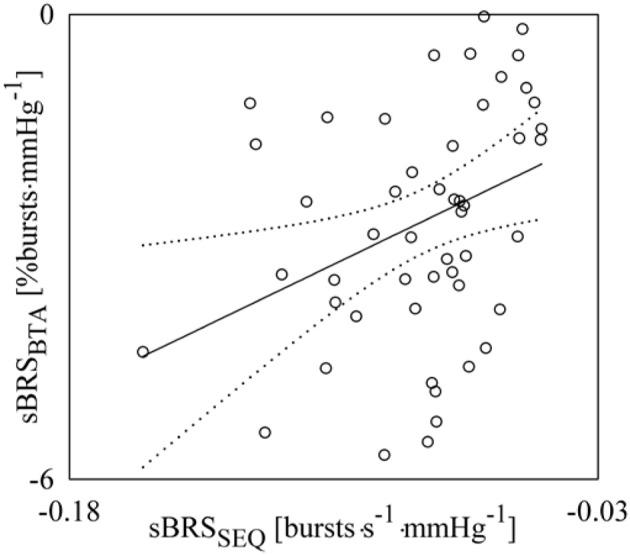
**Linear correlation analysis of sBRS_BTA_ on sBRS_SEQ_ during incremental orthostatic challenge**. Individual pairs (open circles) in the plane (sBRS_SEQ_, sBRS_BTA_) are shown after pooling all subjects together regardless of the experimental condition. The linear regression (solid line) and its 95% confidence interval (dotted lines) are plotted as well if a significant association between the two sBRS measures was found with *p* < 0.05.

Figure 8 shows the pairs (open circles) in the planes (sBRS_SEQ_, sBRS_BTA_) as estimated from each subject regardless of the experimental condition. The regression line of sBRS_BTA_ on sBRS_SEQ_ (solid line) and its 95% confidence interval (dotted lines) are plotted as well if the slope of the regression line is significantly larger than zero. A significant association between sBRS_SEQ_ and sBRS_BTA_ (*r* = 0.343, *p* = 1.36 × 10^−2^) was detected.

**Figure 9:**
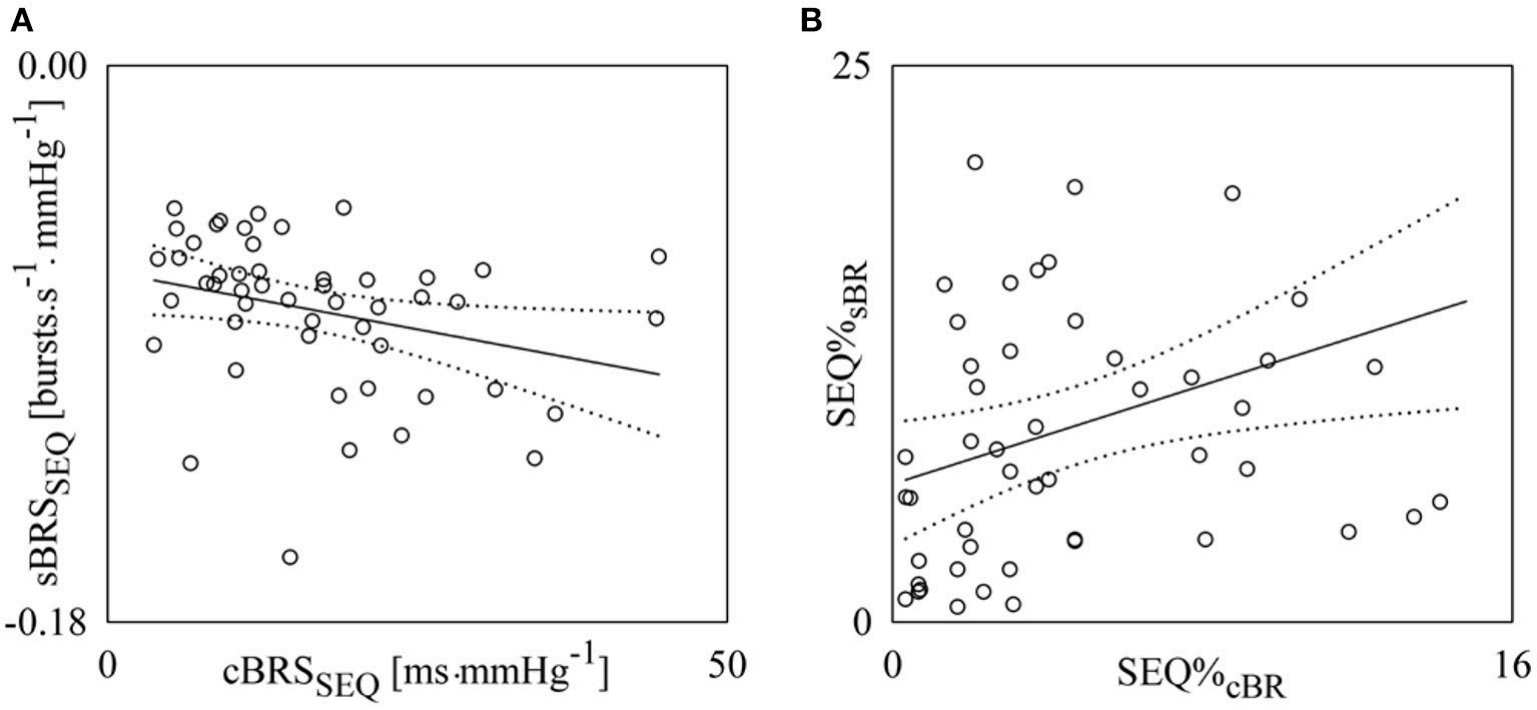
**Linear regression analyses of sBRS_SEQ_ on cBRS_SEQ_ (A) and SEQ%_sBR_ on SEQ%_cBR_ (B) during incremental orthostatic challenge. Individual pairs (open circles) in the plane (cBRS_SEQ_, sBRS_SEQ_) and (SEQ%_*cBR*_,SEQ%_sBR_) are shown after pooling all subjects together regardless of the experimental condition**. The linear regression (solid line) and its 95% confidence interval (dotted lines) are plotted as well if the slope of the regression line is significantly larger than zero with *p* < 0.05.

Figure 9 shows the pairs (open circles) in the planes (cBRS_SEQ_, sBRS_SEQ_) (Figure 9A) and (SEQ%_cBR_, SEQ%_sBR_) (Figure 9B) as estimated from each subject regardless of the experimental condition. The regression lines of sBRS_SEQ_ on cBRS_SEQ_ and SEQ%_sBR_ on SEQ%_cBR_ (solid line) and their 95% confidence interval (dotted lines) are plotted as well because both the slopes of the regression lines are significantly larger than zero. sBRS_SEQ_ was significantly and negatively related to cBRS_SEQ_ (Figure 9A, *r* = −0.302, *p* = 3.30 × 10^−2^), while SEQ%_sBR_ was significantly and positively associated with SEQ%_cBR_ (Figure 9B, *r* = 0.340, *p* = 1.57 × 10^−2^).

## Discussion

The main findings of this study can be summarized as follows: (i) we proposed a spontaneous sBR sequence method exploiting a previously defined MSNA variability series describing the fluctuations of MSNA burst rate about its mean value; (ii) we utilized an incremental head-up tilt protocol to optimize the parameters necessary to detect sBR sequences and we confirmed that the involvement of sBR increased with tilt table angles, while its effectiveness decreased (i.e., SEQ%_sBR_ increased and sBRS_SEQ_ became less negative); (iii) in addition to the sBR we characterized cBR using the cBR sequence method and we confirmed that the involvement of the cBR increased with the magnitude of the orthostatic challenge and its effectiveness gradually declined (i.e., SEQ%_cBR_ increased and cBRS_SEQ_ decreased toward zero); (iv) sBRS_SEQ_ was correlated with that computed via a more traditional static approach (i.e., BTA); (v) cBRS_SEQ_ and sBRS_SEQ_ were significantly correlated as well as SEQ%_cBR_ and SEQ%_sBR_ even though both correlations were weak, thus suggesting that the cBR and sBR controls exhibit a certain degree of independence, while they are both activated in relation to the magnitude of the postural challenge.

### Spontaneous sBR sequence method

Traditionally the sBR was assessed via the modified Oxford method (Rudas et al., 1999; Studinger et al., 2009; Dutoit et al., 2010; Hart et al., 2010). This pharmacological method imposes a rapid and wide modification of AP by administering in rapid succession a depressor agent (i.e., sodium nitroprusside) followed by a pressor one (i.e., phenylephrine). The modified Oxford method, originally devised for the characterization of the cBR (Ebert and Cowley, 1992), assesses sBR in nonphysiological conditions evoked by the administration of vasoactive drugs. In the attempt to overcome this issue, a static method based on spontaneous MSNA and DAP fluctuations has been devised (Sundlöf and Wallin, 1978; Kienbaum et al., 2001; O'Leary et al., 2003; Fu et al., 2006; Ichinose et al., 2006; Keller et al., 2006; Hart et al., 2010, 2011). The nonpharmacological method is grounded on counting the percentage of MSNA bursts associated with binned values of DAP observed during spontaneous fluctuations of AP and on the assessment of the linear relation describing the progressive inhibition of MSNA with DAP. In addition to the avoidance of the administration of the pharmacological stimulus, this method has an additional advantage: indeed, given the smallness of spontaneous physiological fluctuations of DAP, the hypothesis of linearity of the relation between AP and MSNA is more likely to be fulfilled compared to the pharmacological method. The potential drawback of the nonpharmacological method lies in its inability to assure the sole exploration of the causal link from DAP to MSNA. Indeed, since it is based on a static association of MSNA bursts with DAP values, the high MSNA-DAP correlation might not be the effect of dynamical variations of DAP (i.e., rises or falls) followed by the appropriate MSNA burst rate change compatible with a sBR origin (i.e., MSNA burst rate decreases or increases, respectively). Another potential drawback of the nonpharmacological approach is that it requires relatively long recordings to guarantee that a sufficient number of MSNA bursts can be associated with any bin of DAP.

In this study we propose a method exploiting the spontaneous fluctuations of MSNA burst rate and DAP about their mean values. The method is inspired by the cBR sequence technique frequently utilized to assess cBR via the detection of SAP and HP sequences featuring contemporaneous increases or decreases of both variables (Bertinieri et al., 1985). By analogy with the cBR sequence technique, the sBR sequence method is based on the search of spontaneous sequences in MSNA and DAP series characterized by the contemporaneous decrease of DAP and increase of MSNA burst rate or *vice versa*. The sBR sequence method features two relevant advantages compared to the nonpharmacological methods (Sundlöf and Wallin, 1978; Kienbaum et al., 2001; Hart et al., 2010): (i) it is fully causal, because it is explicitly based on the detection of sequences that can be exclusively attributed to the sBR, thus avoiding the confounding factor related to the association of MSNA with DAP values when their variations are incompatible with a working sBR; (ii) since, in principle, it is sufficient to find a unique sBR sequence to compute the sBRS, this method can be applied to very short recordings of MSNA and DAP. The proposed approach was made possible by the exploitation of a recently defined MSNA variability quantifying the modulations of the MSNA burst rate about its mean (Marchi et al., 2016). The dimensionality of the recently proposed calibrated MSNA variability signal (i.e., bursts^.^s^−1^) allows us to express sBRS with its natural units (i.e., bursts^.^s^−1.^mmHg^−1^), thus facilitating comparison among sBRS values derived from different experimental conditions and groups without requiring normalization procedures. This comparison would be more difficult if the more classical definition of MSNA variability based on low-pass filtering procedure of the integrated MSNA signal preserving the frequency content from 0.0 to 0.5 Hz was utilized (Saul et al., 1990; Pagani et al., 1997; Nakata et al., 1998; Taylor et al., 1998; Cooke et al., 1999; Furlan et al., 2000; Kamiya et al., 2005; Ryan et al., 2011). Indeed, the application of the proposed sBR sequence analysis to the low-pass filtered MSNA variability would lead to express sBRS in less natural units and would require some normalization procedures to avoid the dependence of sBRS on experimental factors such as position of the electrode, number of active units, amplification gain, and effects of noise superposed to the MSNA signal (Marchi et al., 2016). Normalization procedures are applied by traditional static methods for sBR characterization based on the association between amplitude or area of the MSNA burst and DAP value as well (Sundlöf and Wallin, 1978; Kienbaum et al., 2001). Even though the proposed method characterized sBR in the time domain, an alternative sBR characterization in the cardiac beat domain can be easily implemented by following this approach and expressing the MSNA variability in bursts per 100 beats.

### Optimization of the parameters utilized to detect the spontaneous sBR sequences

The minimal |ΔDAP|, being overcome to assure that the input was sufficiently exciting for activating the reflex, was set to 1 mmHg in agreement with the minimal size of the bin utilized to quantize DAP in the nonpharmacological characterization of the sBR (Hart et al., 2010). In addition, this choice has the remarkable advantage of being the most frequent setting for the minimal |ΔSAP| utilized by the cBR sequence analysis (Laude et al., 2004), thus allowing a more homogenous comparison between results derived from the proposed sBR sequence analysis and the traditional cBR sequence method (Bertinieri et al., 1985). The minimal |r_MSNA−DAP_|, being overcome to assure that the degree of MSNA-DAP association was significant over a presumed sBR sequence, was set to 0.85 again in keeping with the similar setting in cBR sequence analysis. The length of the sBR sequence was set to 3 beats according to the minimal number of pairs in the plane [DAP(*i*), MSNA(*i*+τ_MSNA−DAP_)] allowing a meaningful computation of a regression line. A similar setting was adopted in cBR sequence analysis as well (Porta et al., 2000). While the minimal |ΔDAP|, the minimal |r_MSNA−DAP_| and the length of the sBR sequence were chosen according to values present in literature with the main purpose to favor comparison, the minimal |ΔMSNA|, being overcome to assure that the magnitude of the sBR response to |ΔDAP| was significant, and the latency τ_MSNA−DAP_, allowing the most appropriate association of the sensed AP variation with the MSNA burst rate change, were optimized. The parameters were chosen as the ones that allowed us to detect a gradually increasing involvement of sBR control in governing MSNA-DAP variability interactions and a progressive decrease of the effectiveness of the MSNA-DAP relation with the magnitude of the orthostatic stimulus as indicated by the positive relations of SEQ%_sBR_ and sBRS_SEQ_ with the sine of the tilt table angles. Both these relations are expected because they were found using a more traditional nonpharmacological static approach that does not require normalization (i.e., BTA) based on binning DAP values and finding the percentage of MSNA bursts associated with them (Ichinose et al., 2006; Barbic et al., 2015; Marchi et al., 2015). The activation of sBR during a postural challenge prevents the AP drop by inducing peripheral vasoconstriction (Fu et al., 2006), while the reduction of sBRS was mainly attributed to the rise of DAP due to vasoconstriction (Ichinose et al., 2006). |ΔMSNA| > 0.0 bursts^.^s^−1^ and τ_MSNA−DAP_ = 0 beats allowed us to find both relationships to the magnitude of the orthostatic challenge with the strongest degree of association. The setting |ΔMSNA| > 0.0 bursts^.^s^−1^ indicates that there is no particular need to prevent the detection of meaningless variations of MSNA burst rate that, in association with the opposite sign variation of DAP, might produce spurious detections of sBR sequences. This setting might be the consequence of the exploited MSNA variability being inherently unaffected by noise because it is defined as the modulation of the MSNA burst rate about its mean value. We found that any increase of the minimal level of |ΔMSNA| above 0.0 bursts^.^s^−1^ leads to a drop in the number of the detected sBR sequences that reduces the degree of association of SEQ%_sBR_ with the magnitude of the orthostatic challenge. The setting τ_MSNA−DAP_ = 0 beats suggests that the best association of sBRS_SEQ_ and SEQ%_sBR_ with tilt table angles was found when the sBR sequences were built by linking DAP to MSNA burst rate within the same beat (i.e., within a latency smaller than one HP). As shown in Figure 2 this result is compatible with the adopted latency of the sBR. Since the minimum of the MSNA-DAP cross-correlation function in the range of lags from 0 to 3 was found at lag zero in the majority of the analyses (i.e., 86%), τ_MSNA−DAP_ = 0 appears to be reliable during the overall experimental protocol.

### sBR sequence method vs. BTA approach

BTA method estimates the linear relation of the percentage of MSNA bursts associated with a given binned DAP value on DAP values. This static approach is completely different from the dynamical technique here proposed. Indeed, the sBR sequence approach links a DAP change to a variation of the MSNA burst rate only if their signs are opposite, while in the BTA method MSNA burst occurrence and DAP values are associated regardless of whether variations of DAP produce the opposite changes in the probability of detecting a MSNA burst. Despite this structural difference between the sBR sequence method and BTA approach, we found that sBRS_SEQ_ and sBRS_BTA_ were significantly and positively correlated. This association might be the result of their common features: indeed, both the methods work on the rate of appearance of the MSNA burst and do not require normalization. However, the level of association is not strong, thus suggesting that the two sBRS estimates should not be considered interchangeable and, conversely, they should be regarded as describing different aspects of the same reflex.

### Spontaneous cBR during incremental head-up tilt

Graded head-up tilt induces a gradual sympathetic activation and vagal withdrawal occurring in response to baroreflex unloading associated to central hypovolemia (Montano et al., 1994; Cooke et al., 1999; Furlan et al., 2000; O'Leary et al., 2003; Kamiya et al., 2005; Fu et al., 2006; Porta et al., 2007; Lambert et al., 2008; Baumert et al., 2011; El-Hamad et al., 2015; Marchi et al., 2016; Porta et al., 2016). The modification of the state of the autonomic nervous system in response to head-up tilt with inclination lower than or equal to than 60° produced a significant tachycardia with limited variations of AP (Bahjaoui-Bouhaddi et al., 2000; O'Leary et al., 2003; Fu et al., 2006; Porta et al., 2011). Our time domain parameters were in agreement with the classical characterization of cardiovascular variables during graded head-up tilt: indeed, the increase of μ_MSNA_ indicates a tendency toward a tonic sympathetic activation leading to a progressive reduction of μ_HP_, while μ_SAP_ and μ_DAP_ were preserved. The progressive increase of $\sigma_{\text{MSNA}}^{2}$ with the tilt table inclination indicates that the magnitude of the fluctuations of the MNSA burst rate about its mean value increased and this rise is likely to be responsible for the increase of $\sigma_{\text{SAP}}^{2}$ and $\sigma_{\text{DAP}}^{2}$ (Marchi et al., 2016). Our findings relevant to SEQ%_cBR_ and cBRS_SEQ_ confirm well-known results present in literature and support the strong involvement of the cBR in governing the adjustment of cardiovascular variables. Conclusions held regardless the strategy followed to set τ_HP−SAP_ (i.e., τ_HP−SAP_ = 0 or the τ_HP−SAP_ optimization according to the maximum of the HP-SAP cross-correlation). Indeed, the gradual increase of SEQ%_cBR_ with tilt table inclination is in agreement with the results reported in Bahjaoui-Bouhaddi et al. (2000), Porta et al. (2016) and with the rise of the strength of the HP-SAP association along the temporal direction from SAP to HP reported in Porta et al. (2011). The progressive decrease of cBRS_SEQ_ with tilt table angles is in agreement with results given in Cooke et al. (1999), Bahjaoui-Bouhaddi et al. (2000), O'Leary et al. (2003), Porta et al. (2016) indicating that the more and more increased involvement of the cBR in governing HP-SAP variability interactions with the magnitude of the postural challenge is accompanied by the progressive decline of the effectiveness of this reflex. The reduction of cBRS_SEQ_ is likely to be related to the reduction of HP in presence of virtually unmodified SAP values and to the reduced HP variation resulting from the vagal withdrawal in presence of an increased SAP variability linked to the increased modulation of the sympathetic discharge.

### The simultaneous characterization of cBR and sBR

cBR and sBR are arms of the same negative neural control feedback aiming at confining disproportionate variability of AP inside a more physiological range through the modulation of HP and sympathetic outflow, respectively (Sundlöf and Wallin, 1978; Hunt et al., 2001; Robertson et al., 2012). Although, the two arms share the same finalistic aim (i.e., controlling systemic AP), sensing areas (mainly carotid sinuses and aortic arch), sensed variable (i.e., systemic AP) and receptors (i.e., the high pressure baroreceptors responding to vascular wall stretch), it can be hypothesized that the two reflexes are not fully redundant (Taylor et al., 2015). Indeed, since the target variable, the efferent pathways and the levels of integration are diverse, cBR, and sBR can take care of specific aspects of the baroreflex control. In agreement with these observations we found a significant relation between sBRS_SEQ_ and cBRS_SEQ_ but the detected degree of association cannot be considered strong (i.e., *r* = −0.302, *p* = 3.30 × 10^−2^), thus indicating a certain degree of independence between sBR and cBR controls. This finding stresses the relevance of the contemporaneous monitoring of sBR and cBR to achieve a more complete and insightful characterization of the human baroreflex regulation. This observation is in agreement with (Taylor et al., 2015) who found a significant relation between cBRS and sBRS in healthy subjects at rest in supine condition using methods based on spontaneous variability (i.e., BTA and cBR sequence technique). The present study extends the results reported in Taylor et al. (2015) in situations of mild sympathetic activation such as those induced by head-up tilt with small tilt table inclinations. This conclusion is in disagreement with the results reported in Rudas et al. (1999) and Dutoit et al. (2010) reporting the uncorrelation between sBRS and cBRS. The origin of the disagreement might be related to the approach followed in Rudas et al. (1999) and Dutoit et al. (2010) who computed sBRS and cBRS according to pharmacological methods (i.e., the modified Oxford technique) producing results that are hardly comparable with those based on spontaneous fluctuations (Taylor et al., 2015). The presence of a significant correlation between SEQ%_cBR_ and SEQ%_sBR_, suggests that both sBR and cBR controls were simultaneously activated in proportion to the challenge. Remarkably, the number of detected cBR and sBR sequences was small compared to the total amount of sequences (i.e., SEQ%_cBR_ and SEQ%_sBR_ were on average < 5 and 9%, respectively), thus indicating that cBR and sBR, even though relevant cardiovascular regulations, operate with several other different mechanisms contributing to adjust HP and MSNA burst rate according to different logics.

### Limitations of the study and future developments

The procedure exploited to set the parameters for the detection of sBR sequences is inherently based on the hypothesis of a positive relation of SEQ%_sBR_ and sBRS_SEQ_ with tilt table inclination in keeping with, respectively, a more and more important involvement of sBR control and a reduction of the magnitude of its intervention with the relevance of the baroreflex unloading (Ichinose et al., 2006; Barbic et al., 2015; Marchi et al., 2015). While there is a broad agreement in the literature about the increased involvement of sBR control with the magnitude of the baroreflex unloading (O'Leary et al., 2003; Fu et al., 2006; Ichinose et al., 2006; Barbic et al., 2015), the reduction of sBRS with tilt table inclination is much more controversial. For example, there are some studies reporting more negative values of sBRS during head-up tilt compared to T0 (O'Leary et al., 2003; Fu et al., 2006). In our data sBRS was neither more negative during head-up tilt compared to T0 nor negatively related to tilt table angles. Conversely, our data supported the presence of a significant positive relation of sBRS with the magnitude of the orthostatic challenge. It is worth noting that in Fu et al. (2006) and O'Leary et al. (2003) nonpharmacological methods expressing sBRS in arbitrary units and requiring normalization were utilized. Future studies should test whether increasing further the relevance of the orthostatic stimulus by exposing the subject to tilt table inclinations of 75 and 90° could reduce even further the sBRS. In addition, it remains to be established whether conclusions might depend on the selection of the sensed variable (e.g., the use of DAP in the study of sBR and of SAP in the study of cBR). As a further limitation this study did not face the issue of the sparseness of MSNA and its possible effect on the sBR sequence method. We advocate a specific evaluation of this issue by explicitly comparing the characterization of sBR in a group of subjects with sparse MSNA and a group with a higher tonic MSNA within the same experimental condition (e.g., during T0). Moreover, since this study is focused on methods of sBR characterization that do not require normalization because they are based on counting MSNA bursts and assessing its rate of occurrence such as the BTA and sBR sequence techniques, their eventual link with those requiring normalization procedures because they are based on MSNA low-pass filtering focusing the frequency content between 0.0 and 0.5 Hz (Saul et al., 1990; Pagani et al., 1997; Nakata et al., 1998; Taylor et al., 1998; Cooke et al., 1999; Furlan et al., 2000; Kamiya et al., 2005; Ryan et al., 2011) or on the evaluation of the amplitude or area of the MSNA bursts (Sundlöf and Wallin, 1978; Kienbaum et al., 2001) requires further studies. Another issue that deserves a specific study featuring a larger number of subjects and, thus, a higher statistical power, is the comparison between the characterization of cBR and sBR within a given experimental condition.

#### Perspectives and significance

We proposed a causal method for the characterization of the sBR from spontaneous beat-to-beat variability of MSNA burst rate and DAP based on the definition and extraction of sequences of sBR origin. Since this proposed method follows the same logic as the cBR sequence technique, the contemporaneous exploitation of both techniques allows one to set a framework assuring a homogenous characterization of both cBR and sBR, thus favoring studies aiming at understanding cardiovascular control and the degree of independence between the two baroreflex arms, e.g., in heart failure patients (Watson et al., 2007). In addition, the proposed method allows one to overcome some limitations of more traditional estimates of sBRS such as the administration of a pharmacological challenge in pharmacological approaches, the inherent need of long recordings in nonpharmacological applications, and the necessity of normalization procedures typical of methods based on computation of amplitude or area of the MSNA bursts.

## Author contributions

AM analyzed the data; AM and AP drafted the manuscript; ME and EL performed the experiments; AM, VB, BD, ME, EL, MB, and AP interpreted the data; AM, VB, BD, ME, EL, MB, and AP revised the manuscript; AM, VB, BD, ME, EL, MB, and AP approved the final version of the manuscript.

### Conflict of interest statement

The authors declare that the research was conducted in the absence of any commercial or financial relationships that could be construed as a potential conflict of interest.

## Abbreviations

sBR: sympathetic baroreflex
cBR: cardiac baroreflex
sBRS: sBR sensitivity
cBRS: cBR sensitivity
sBRS_SEQ_: sBRS computed via the sBR sequence method
SEQ%_sBR_: percentage of sBR sequences
BTA: baroreflex threshold analysis
sBRS_BTA_: sBRS computed via the BTA method
cBRS_SEQ_: cBRS computed via the cBR sequence method
SEQ%_cBR_: percentage of cBR sequences
ECG: electrocardiogram
AP: arterial pressure
HP: heart period
SAP: systolic AP
DAP: diastolic AP
MSNA: muscle nerve sympathetic activity
r: correlation coefficient
p: type I error probability
τ: lag between physiological variables expressed in beats
T0, T20, T30, T40, T60: head-up tilt with table inclination of 0, 20, 30, 40, and 60°.
